# The joint effect of maternal smoking during pregnancy and maternal pre-pregnancy overweight on infants’ term birth weight

**DOI:** 10.1186/s12884-020-2816-3

**Published:** 2020-02-27

**Authors:** Thanin Chattrapiban, Henriette A. Smit, Alet H. Wijga, Bert Brunekreef, Judith M. Vonk, Ulrike Gehring, Lenie van Rossem

**Affiliations:** 1 Julius Center for Health Sciences and Primary Care, University Medical Center Utrecht, Utrecht University, Universiteitsweg 100, STR 6.118, Utrecht, 3508 GA The Netherlands; 2 Center for Prevention and Health Services Research, National Institute of Public Health and the Environment, P.O Box 1, 3720 BA Bilthoven, The Netherlands; 3 Institute for Risk Assessment Science (IRAS), Utrecht University, Yalelaan 2, 3584 CM Utrecht, The Netherlands; 4 Department of Epidemiology, University of Groningen, University Medical Center Groningen, Hanzeplein 1, 9713 GZ Groningen, The Netherlands; 5 Department of Obstretrics and Gynecology, Erasmus MC University Medical Center, Dr. Molewaterplein 50, 3015 GD Rotterdam, the Netherlands

**Keywords:** Smoking, Overweight, Pregnancy, Birth weight, Interaction

## Abstract

**Background:**

It is well known that maternal smoking during pregnancy and maternal pre-pregnancy overweight have opposite effects on the infants’ birth weight. We report on the association of the combination between both risk factors and the infants’ birth weight.

**Methods:**

We studied 3241 infants born at term in the PIAMA birth cohort. Maternal smoking during pregnancy and pre-pregnancy height and weight were self-reported. Multivariable regression analysis was performed to assess the associations between infants of mothers who only smoked during pregnancy, who only had pre-pregnancy overweight and who had both risk factors simultaneously, on term birth weight and the risk of being SGA or LGA.

**Results:**

Of 3241 infants, 421 infants (13%) were born to smoking, non-overweight mothers, 514 (15.8%) to non-smoking, overweight mothers, 129 (4%) to smoking and overweight mothers and 2177 (67%) to non-smoking, non-overweight mothers (reference group). Infants of mothers who smoked and also had pre-pregnancy overweight had similar term birth weight (− 26.6 g, 95%CI: − 113.0, 59.8), SGA risk (OR = 1.06, 95%CI: 0.56, 2.04), and LGA risk (OR = 1.09, 95%CI: 0.61, 1.96) as the reference group.

**Conclusions:**

Our findings suggested that the effects of maternal smoking during pregnancy and maternal pre-pregnancy overweight on infants’ birth weight cancel each other out. Therefore, birth weight may not be a good indicator of an infant’s health status in perinatal practice because it may mask potential health risks due to these maternal risk factors when both present together.

## Background

Maternal smoking during pregnancy and pre-pregnancy overweight are well-known examples of obstetric risk factors that have an impact on infants’ health [1–5]. Infants of mothers who smoked during pregnancy have on average a lower birth weight and a greater risk of being small-for-gestational age (SGA) than those born to non-smoking mothers [1, 2, 4, 6]. In contrast, infants of pre-pregnancy overweight mothers have a higher birth weight and a greater risk of being large-for-gestational age (LGA) than those of normal-weight mothers [3, 5, 7, 8].

Relatively little is known about the combined effect of maternal smoking during pregnancy and maternal pre-pregnancy overweight on birth weight. The individual, opposite effects of maternal smoking and maternal overweight may either cancel each other out resulting in normal birth weight as was observed in a recent large population-based study [9], or the individual effect of one maternal risk factor may prevail over the other as was reported in a less recent, smaller study [10]. Birth weight is often used as an indicator of the infant’s health status which may not be valid for these common risk factors with opposite effects.

Therefore, the present study examined the combined effect of maternal smoking during pregnancy and pre-pregnancy overweight on term birth weight, the risk of being born SGA or LGA in the PIAMA birth cohort, a prospective birth cohort in the Netherlands.

## Methods

### Study design and setting

The present study is part of a population-based Dutch birth cohort study: The Prevention and Incidence of Asthma and Mite Allergy (PIAMA) Study. A detailed description of the study design has previously been published [11]. In brief, mothers were recruited from the general population during pregnancy and the children were born in 1996–1997. The baseline population consisted of 3963 children. The study protocol was approved by the medical ethics committees of the participating institutes and all parents gave written informed consent.

### Study population

Of 3963 infants, we excluded 190 infants who were born < 37 weeks of gestation as well as 33 infants with missing information on gestational age. We additionally excluded infants with missing values on maternal smoking during pregnancy (*n* = 33), maternal pre-pregnancy overweight (*n* = 451), and birth weight (*n* = 15) from the analysis. Therefore, the population for analysis consisted of 3241 infants.

### Data collection

In the PIAMA study, data were collected by self-administered questionnaires which were sent to the parents during pregnancy, at 3 months after birth, yearly from the child’s age of 1 to 8 years, and at 11, 14 and 17 years. The present study used data collected during pregnancy, at 3 months after birth and at the child’s age of 1 and 2 years. Data on maternal smoking were collected during pregnancy. Maternal age (years) and parity was assessed by questionnaire at 3 months after the child’s birth. Data on maternal educational level, weight and height before pregnancy were collected in the 1-year questionnaire and maternal ethnicity in the 2-year questionnaire.

### Definition of variables

#### Exposures and outcomes

Maternal smoking during pregnancy was categorized into smoking and non-smoking and refers to those who reported smoking during at least the first 4 weeks of pregnancy. This variable was created based on information on current smoking during pregnancy and on information on timing of quitting in mothers who reported to be former smokers. Information on current smoking status, smoking intensity and months since quitting was assessed at the time of completing the ‘pregnancy questionnaire’: 95% of the ‘pregnancy’ questionnaires were completed in the 3rd trimester of pregnancy (mean 33 wks; SD 3). Smoking intensity was asked only if mothers were still smoking at the time of completion of the questionnaire, and represent only the number of cigarettes they were smoking at that time. Maternal pre-pregnancy body mass index (BMI) was calculated as body weight (kg) divided by height squared (m^2^). We dichotomized pre-pregnancy maternal BMI into overweight and obesity (BMI ≥ 25 kg/m^2^) and non-overweight (BMI < 25 kg/m^2^). With these two binary variables of maternal smoking and maternal overweight, infants were classified into four groups that will be referred to as: infants who were born to (i) non-smoking, non-overweight (the reference group), (ii) smoking, non-overweight, (iii) non-smoking overweight, and (iv) smoking, overweight mothers.

The primary outcome was term birth weight (grams). In the 3-months questionnaire, parents were asked to report their infant’s birth weight and gestational age from the delivery report. SGA was defined as infants with birth weight below the 10th percentile for gestational age, and LGA was defined as infants with birth weight above the 90th percentile for gestational age according to the standard national growth curve taking into account the infant’s sex and parity [12].

#### Covariates

Maternal age, ethnicity, educational level and parity (parity for birth weight only) were considered as potential confounders which were set at priori based on literature [13]. Maternal ethnicity was classified according to birth country as Dutch; western, non-Dutch (Europe (except Turkey), North-America, Oceania, Indonesia, Japan); and non-western (Africa, Latin-America, Asia (except Indonesia, Japan), Turkey). Educational level was categorized into three levels: low (primary schools, low vocational training, or lower secondary education), intermediate (intermediate vocational training, or intermediate/higher secondary education), and high (higher vocational training or university degree). The presence of older siblings was used as a proxy for parity and categorized into two groups: none and one or more. Gestational age was measured in weeks.

### Statistical analysis

All statistical analyses were performed with Stata statistical software version 12.1 (Stata Corporation, TX). Statistical significance was defined by a two-sided alpha-level *p* < 0.05. Baseline characteristics of study participants were reported according to the four exposure categories. Differences in proportions for categorical variables were evaluated using Chi square or Fisher’s exact tests. Means with standard deviations were used to describe the distribution of continuous variables. One-way ANOVA with Bartlett’s test for equal variances was used to test the difference in means between exposure groups.

Multivariable linear regression analysis was performed to examine the association of maternal smoking during pregnancy, pre-pregnancy overweight, and the combination thereof on term birth weight. The beta-coefficients represent the difference in term birth weight between infants with the exposure of interest and the reference category. Multivariable logistic regression analysis was used to assess the associations of maternal risk factors with the risk of being SGA or LGA. Associations are reported as odds ratios (ORs) with 95% confidence intervals. All potential confounders were included in the full model, then backward elimination was performed to exclude non-significant confounders one at a time until all variables in the model had a *p*-value of < 0.20. This conservative level was chosen to be sure that no confounders were missed.

#### Stratified and joint analyses

After assessing the main effects of maternal smoking and maternal pre-pregnancy overweight, we performed stratified analyses to assess the presence of effect modification. We examined whether (1) the association between maternal smoking during pregnancy and infants’ term birth weight, and the risk of being SGA is modified by maternal pre-pregnancy overweight, (2) the association between maternal pre-pregnancy overweight and birth weight, and the risk of being LGA is modified by maternal smoking during pregnancy.

To assess the joint effect of maternal smoking during pregnancy and maternal pre-pregnancy overweight with 95% confidence intervals, we used a 4-category exposure variable (infants of non-smoking, non-overweight mothers, smoking, non-overweight mothers, non-smoking, overweight mothers and smoking, overweight mothers) in the regression models with adjustment for the same set of confounders using infants of non-smoking, non-overweight mothers as the reference category. The presence of interaction was not tested statistically due to a lack of power.

### Sensitivity analyses

We performed three types of sensitivity analyses and we presented the results in the supplement. First, we performed a sensitivity analysis to assess whether the severity of maternal pre-pregnancy overweight changed the estimates and affected our interpretation. We therefore repeated our analyses with three categories of maternal weight: non-overweight, overweight (BMI 25–30 kg/m^2^), and obese (BMI ≥ 30 kg/m^2^). Second, we performed a sensitivity analysis taking smoking intensity into account. We expressed smoking intensity (number of cigarettes per day) as a categorical variable. Infants of non-smoking mothers formed the reference category, and infants of smoking mothers were categorized in quartiles of the numbers of cigarettes per day: > 0–2, 3–5, 6–10, and > 10 cig/d. Information on smoking intensity was available for mothers who were current smokers at the time of filling in the ‘pregnancy questionnaire’ which was in the 3rd trimester of pregnancy for 95% of the mothers. Since we defined our maternal smoking variable as smoking during ‘at least the first 4 weeks of pregnancy’, and since 22% of the smoking mothers had quit by the time of filling the ‘pregnancy questionnaire’, we have data on smoking intensity of 78% (429/550) of the infants with smoking mothers. To compare the effect estimates of maternal smoking intensity on birth weight with the effect estimates of the dichotomous smoking variable on birth weight, we repeated the main analysis (Table 2) using the study population excluding the 121 infants of smoking mothers without information on smoking intensity. The third sensitivity analysis was performed to explore the effect of adjustment for gestational weight gain (GWG) in the association under study. We expressed GWG as a continuous variable (kg) and as a categorical variable with cut-off points based on the guideline of the US Institute of Medicine [14]: inadequate, adequate and excessive weight gain. We repeated the main analysis additionally adjusted for GWG and added the results of the joint effect in the supplementary tables. Due to small numbers, we were not able to stratify on GWG and disentangle the independent effect of GWG and pre-pregnancy overweight.

## Results

### Characteristics of the study population

Of 3241 infants born at term, 421 infants (13%) were born to smoking, non-overweight mothers, 514 (15.8%) to non-smoking, overweight mothers, 129 (4%) to smoking and overweight mothers. The reference group consisted of 2177 (67.2%) infants who were born to non-smoking, non-overweight mothers. The characteristics of the study population are described according to these categories in Table 1.

**Table 1:** Characteristics of the study population by category of maternal exposure

Characteristics	All (*n* = 3241)	Non-smoking mothers		Smoking mothers	
		Non-overweight mothers (*N* = 2177)	Overweight mothers (*N* = 514)	Non-overweight mothers (*N* = 421)	Overweight mothers (*N* = 129)
	% (n)	% (n)	% (n)	% (n)	% (n)
Maternal age (years)*					
< 25	5.7 (185)	4.9 (106)	4.9 (25)	9.8 (41)	10.2 (13)
25–34	79.5 (2566)	79.5 (1725)	83.2 (425)	75.4 (316)	78.1 (100)
≥ 35	14.8 (478)	15.6 (340)	11.9 (61)	14.8 (62)	11.7 (15)
Mean (SD)	30.5 (3.8)	30.7 (3.7)	30.0 (3.8)	30.0 (4.2)	29.5 (3.9)
Maternal ethnicity					
Dutch	95.8 (3004)	95.7 (2022)	96.0 (486)	96.0 (384)	94.1 (112)
Western, non- Dutch	2.6 (82)	2.8 (59)	1.8 (9)	2.3 (9)	4.2 (5)
Non-western	1.6 (51)	1.5 (31)	2.2 (11)	1.7 (7)	1.7 (2)
Education level*					
Low	21.8 (707)	16.3 (355)	27.6 (142)	34.4 (144)	51.2 (66)
Intermediate	41.6 (1347)	41.3 (898)	45.4 (233)	40.1 (168)	37.2 (48)
High	36.6 (1183)	42.4 (922)	27.0 (139)	25.5 (107)	11.6 (15)
Presence of older siblings					
None	48.6 (1575)	49.1 (1069)	47.9 (246)	47.7 (201)	45.7 (59)
1 or more	51.4 (1666)	50.9 (1108)	52.1 (268)	52.3 (220)	54.3 (70)
Gestational weight gain (kg)**					
Mean (SD)	13.8 (4.9)	13.7 (4.4)	12.9 (5.5)	15.5 (5.6)	13.3 (7.5)
Birth weight (g)**					
Mean (SD)	3560 (482)	3567 (469)	3678 (472)	3390 (500)	3521 (524)
SGA*					
Yes	7.7 (248)	7.2 (157)	4.3 (22)	13.9 (58)	8.7 (11)
No	92.3 (2978)	92.8 (2011)	95.7 (491)	86.1 (360)	91.3 (116)
LGA*					
Yes	11.3 (364)	10.5 (228)	17.4 (89)	7.9 (33)	11.0 (14)
No	88.7 (2862)	89.5 (1940)	82.6 (424)	92.1 (385)	89.0 (113)
Child’s gender					
Girl	48.5 (1573)	48.1 (1048)	47.9 (246)	52.5 (221)	45.0 (58)
Boy	51.5 (1668)	51.9 (1129)	52.1 (268)	47.5 (200)	55.0 (71)

*Abbreviation*s *SGA* Small for gestational age, *LGA* Large for gestational age

* *P*-value < 0.05 by Fisher’s exact test

** *P*-value < 0.05 by oneway ANOVA

In infants of mothers who smoked during pregnancy (irrespective of pre-pregnancy overweight) the mothers more often were younger than 25 years, compared to the reference group (9.8 and 10.2% versus 4.9% respectively). In infants of mothers who smoked and also had pre-pregnancy overweight, the mothers more often had a low educational level.

### Association of maternal smoking during pregnancy with term birth weight and SGA in strata of maternal pre-pregnancy overweight

In the overall analysis on the association of maternal smoking during pregnancy with birth weight and SGA, infants of smoking mothers had a lower term birth weight than those of non-smoking mothers, with an adjusted mean difference in birth weight of − 158.4 g (95%CI: − 203.4, − 113.4). The lower birth weight was of similar magnitude in both strata of maternal pre-pregnancy overweight with an adjusted mean difference in birth weight of − 164.5 g (95%CI: − 215.2, − 113.8) in the stratum without maternal pre-pregnancy overweight and − 145.2 g (95%CI: − 241.1, − 49.3) in the stratum with maternal pre-pregnancy overweight (Table 2).

**Table 2:** Associations of maternal smoking during pregnancy with term birth weight and the risk of being SGA: overall and in strata of maternal pre-pregnancy overweight

	Birth weight (g)		SGA		
	Crude Diff (g) (95% CI)	Adjusted Diff ^a^ (g) (95% CI)	SGA/Total (%)	Crude OR (95% CI)	Adjusted OR ^b^ (95% CI)
Main effect					
Non-smoking	0 (Ref)	0 (Ref)	179/2681 (6.7%)	1.0 (Ref)	1.0 (Ref)
Smoking	− 167.6 (− 211.5, − 123.7)	−158.4 (−203.4, −113.4)	69/545 (12.7%)	2.02 (1.51, 2.72)	1.92 (1.42, 2.60)
Strata of maternal pre-pregnancy overweight:					
Non-overweight					
Non-smoking	0 (Ref)	0 (Ref)	157/2168 (7.2%)	1.0 (Ref)	1.0 (Ref)
Smoking	−177.1 (− 226.6, − 127.6)	−164.5 (−215.2, −113.8)	58/418 (13.9%)	2.06 (1.49, 2.85)	1.96 (1.41, 2.72)
Overweight					
Non-smoking	0 (Ref)	0 (Ref)	22/513 (4.3%)	1.0 (Ref)	1.0 (Ref)
Smoking	− 156.9 (− 250.4, −63.5)	−145.2 (−241.1, −49.3)	11/127 (8.6%)	2.12 (0.99, 4.48)	1.89 (0.87, 4.09)

*Abbreviations*: *Diff* mean difference, *SGA* Small for gestational age, *OR* Odds ratio, *CI* Confidence interval

^a^Multivariable linear regression adjusted for maternal age and education

^b^Multivariable logistic regression adjusted for maternal age and education

In the overall analysis on SGA, infants of smoking mothers had a higher risk of being born SGA (adjusted OR = 1.92, 95%CI: 1.42, 2.60) than those of non-smoking mothers. The greater risk of being SGA in infants from smoking versus non-smoking mothers was of similar magnitude in both strata of maternal pre-pregnancy overweight with an adjusted OR of 1.96 (95%CI: 1.41, 2.72) in the stratum without pre-pregnancy overweight and 1.89 (95%CI: 0.87, 4.09) in the stratum with pre-pregnancy overweight (Table 2).

### Association of maternal pre-pregnancy overweight with term birth weight and LGA in strata of maternal smoking during pregnancy

In the overall analysis of the association of maternal pre-pregnancy overweight on birth weight and LGA, infants of pre-pregnancy overweight mothers had a higher term birth weight than those of non-overweight mothers, with an adjusted mean difference in birth weight of 121.8 g (95%CI: 79.7, 163.9). The higher birth weight was of similar magnitude in both strata of maternal smoking during pregnancy with an adjusted mean difference in birth weight of 118.2 g (95%CI: 72.3, 164.1) in the stratum without maternal smoking and 147.7 g (95%CI: 45.7, 249.7) in the stratum with maternal smoking (Table 3).

**Table 3:** Associations of maternal pre-pregnancy overweight with term birth weight and the risk of being LGA; overall and in strata of maternal smoking during pregnancy

	Birth weight (g)		LGA		
	Crude Diff (95% CI)	Adjusted Diff ^a^ (95% CI)	LGA/Total (%)	Crude OR (95% CI)	Adjusted OR ^b^ (95% CI)
Main effect					
Non-overweight	0 (Ref)	0 (Ref)	261/2325 (10.1%)	1.0 (Ref)	1.0 (Ref)
Overweight	107.6 (66.1, 149.1)	121.8 (79.7, 163.9)	103/537 (16.1%)	1.71 (1.34, 2.18)	1.75 (1.36, 2.25)
Strata maternal smoking during pregnancy					
Non-smoking					
Non-overweight	0 (Ref)	0 (Ref)	228/1940 (10.5%)	1.0 (Ref)	1.0 (Ref)
Overweight	110.4 (65.2, 155.6)	118.2 (72.3, 164.1)	89/424 (17.4%)	1.78 (1.37, 2.33)	1.79 (1.37, 2.35)
Smoking					
Non-overweight	0 (Ref)	0 (Ref)	33/385 (7.9%)	1.0 (Ref)	1.0 (Ref)
Overweight	130.6 (30.6, 230.6)	147.7 (45.7, 249.7)	14/113 (11.0%)	1.44 (1.13, 4.88)	1.61 (0.75, 2.79)

*Abbreviations*: *Diff* mean difference, *LGA* Large for gestational age, *OR* Odds ratio, *CI* Confidence interval

^a^Multivariable linear regression adjusted for maternal age and education

^b^Multivariable logistic regression adjusted for maternal age and education

In the overall analysis on LGA, infants of pre-pregnancy overweight mothers had a greater risk of being born LGA (adjusted OR = 1.75, 95%CI: 1.36, 2.25) than those of non-overweight mothers. The greater risk of being LGA in infants from overweight versus non-overweight mothers was of similar magnitude in both strata of maternal smoking during pregnancy where an adjusted OR was 1.79 (95%CI: 1.37, 2.35) in the stratum without maternal smoking and 1.61 (95%CI: 0.75, 2.79) in the stratum with maternal smoking (Table 3).

### Combination of maternal smoking during pregnancy and pre-pregnancy overweight on term birth weight, SGA and LGA

To study the joint effect of maternal smoking and maternal pre-pregnancy overweight on birth weight and SGA or LGA, we focused on infants of mothers with both risk factors (those who smoked during pregnancy and also had pre-pregnancy overweight) using infants of non-smoking, non-overweight mothers as a reference group. Infants of mothers with both risk factors had a similar term birth weight with an adjusted mean difference of − 26.6 g (95%CI: − 113, 59.8) and a similar risk of being SGA (adjusted OR = 1.06, 95%CI: 0.56, 2.04), and LGA (adjusted OR = 1.09, 95%CI: 0.61, 1.96) as infants of mothers without both risk factors (Table 4).

**Table 4:** The effects of the combinations of exposure to maternal smoking during pregnancy and pre-pregnancy overweight on term birth weight and the risk of being SGA and LGA

Categories		Birth weight (g)		SGA		LGA	
		Crude Diff (g) (95% CI)	Adjusted Diff ^a^ (g) (95% CI)	Crude OR (95% CI)	Adjusted OR ^b^ (95% CI)	Crude OR (95% CI)	Adjusted OR ^c^ (95% CI)
**SM -**	**OV-**	0 (Ref)	0 (Ref)	1.0 (Ref)	1.0 (Ref)	1.0 (Ref)	1.0 (Ref)
**SM+**	**OV-**	−177.1 (−226.8, −127.4)	−163.8 (− 214.4, −113.1)	2.06 (1.49, 2.84)	1.94(1.39, 2.69)	0.73 (0.49, 1.07)	0.75 (0.51, 1.09)
**SM -**	**OV+**	110.4 (64.6, 156.2)	120.4 (74.1, 166.8)	0.57 (0.36, 0.90)	0.55 (0.34, 0.86)	1.79 (1.37, 2.33)	1.81 (1.38, 2.37)
**SM+**	**OV+**	−46.6(− 131.2, 38.1)	−26.6(−113.0, 59.8)	1.21 (0.64, 2.30)	1.06 (0.56, 2.04)	1.05 (0.59, 1.87)	1.09 (0.61, 1.96)

*Abbreviations*: *Diff* mean Difference, *SGA* Small for gestational age, *LGA* Large for gestational age, *OR* Odds ratio, *CI* Confidence interval, *SM* maternal smoking during pregnancy, *OV* maternal pre-pregnancy overweight

^a^Multivariable linear regression adjusted for maternal age and education

^b^Multivariable logistic regression adjusted for maternal age and education

^c^Multivariable logistic regression adjusted for maternal age and education

### Sensitivity analysis

In the first sensitivity analysis with three strata of maternal pre-pregnancy BMI (non-overweight, overweight, obese), the lower term birth weight (− 163.2 g, 95%CI: − 394.1, 67.7) and increased risk of being SGA (OR = 1.24, 95%CI: 0.26, 5.99) for infants of smoking mothers was also observed in the stratum of obese mothers, and these effects were not different for obese and overweight mothers (Supplementary Table S1). Also, the effects of maternal pre-pregnancy overweight and obesity were similar in terms of term birth weight, and the risk of being LGA by the strata of maternal smoking (Supplementary Table S2). Infants of mothers who smoked during pregnancy *and* were obese before pregnancy had term birth weight (34.7 g, 95%CI: − 135.9, 205.3), risk of being SGA (OR = 1.13, 95%CI: 0.33, 3.79) and LGA (OR = 2.17, 95%CI: 0.87, 5.44) comparable to those of mothers without these risk factors (Supplementary Table S3). The second sensitivity analysis showed that smoking intensity was somewhat higher in overweight mothers than in non-overweight mothers (Supplementary Table S4). In the overall analysis, and in the stratum of mothers with normal pre-pregnancy weight, infants of mothers who smoked more than 2 cig/d had a lower birth weight than those of non-smoking mothers and mothers who smoked less than 2 cig/d (lowest smoking intensity). However, in the stratum of overweight mothers, the lower birth weight was observed only at the higher smoking intensity of 6 or more cig/day (Supplementary Table S5). Due to small numbers, the confidence intervals around the point estimates were wide and there was no clear dose-response relationship of birth weight with smoking intensity of > 2 cig/day. The third sensitivity analysis (joint effect analysis in supplemental Table S6) suggests that additional adjustment for GWG slightly increased the effect estimates in the same direction as the main analysis (Table 4). In infants of smoking, non-overweight mothers, the effect on lower birth weight and the higher risk of SGA was stronger; in infants of non-smoking, overweight mothers, the effect on higher birth weight and higher risk of LGA was stronger. The combined effect of overweight and smoking became slightly more attenuated to no difference in birth weight and no increased risk of SGA and LGA.

## Discussion

We found that infants of smoking, overweight mothers had an average term birth weight and risk of being born SGA or LGA which was comparable to that of infants of mothers who did not smoke and had no pre-pregnancy overweight. Since we also observed that maternal smoking during pregnancy was significantly associated with a lower term birth weight and a higher risk of being born SGA; maternal pre-pregnancy overweight was significantly associated with a higher term birth weight and a higher risk of being born LGA. This indicates that the effects of maternal smoking during pregnancy and of maternal pre-pregnancy overweight on term birth weight and the risk of SGA and LGA cancel each other out.

### Methodological considerations

The large size of the study population allowed infants to be cross-classified based on maternal smoking and maternal overweight so that we could examine the joint effect of both risk factors. The PIAMA study is an unselected cohort, but due to lower non-response and selective loss-to-follow-up, infants of highly educated, more urban and allergic mothers were overrepresented in the cohort. However, even if the distribution of pre-pregnancy overweight and smoking during pregnancy differs between infants of high and low educated, urban and rural or between allergic and non-allergic mothers there is no firm ground to argue that the association between exposure and birth weight also differs between these groups. Therefore the observed association can be generalized to the general population of infants of western origin. We also emphasize that the results cannot be generalized towards children who were born prematurely since we restricted our analyses to infants born at full-term. The prevalence of preterm births in the PIAMA study (4.2%) was comparable to that in the general population (approximately 5–9%), [15] but it was too low to allow for separate analyses within this group. Some methodological limitations need to be considered. First, we assessed smoking habits and pre-pregnancy BMI status by self-reporting which could have introduced bias if for example true smoking habits were underreported by smokers and BMI status (height and weight) was underestimated by overweight women. It is likely that this misclassification bias is random and underestimates rather than overestimates the observed effect size of each individual maternal risk factor on birth weight, risk of SGA and LGA. Second, the influences of unmeasured confounders such as genetic and environmental factors cannot be ruled out in the association under study. However, since maternal smoking during pregnancy and pre-pre-pregnancy overweight are known strong predictors for infant’s birth weight, we assume that the contribution of these confounders is trivial. Thirdly, missing pre-pregnancy BMI data could have attenuated the effect size of maternal pre-pregnancy overweight on birth weight and LGA risk. This holds true only if the missingness is related to the outcome (i.e. mothers who had heavy babies did not report their pre-pregnancy BMI). However, we believe that the missing BMI data would not largely bias our results because it was not extensive (~ 11%; 451 of 3963) and in addition the effect of maternal pre-pregnancy overweight observed in our complete case analysis are consistent to that reported in a recent systematic review with the same interpretation [8]. A fourth issue is the availability of more detailed information on maternal smoking during pregnancy. In this study, we investigated the effect of absence and presence of smoking during pregnancy as a dichotomous variable but we realize that also the timing, duration and intensity of smoking during pregnancy may determine the effect on birth weight. We assessed maternal smoking at one point in time mostly in the third trimester of pregnancy. However, due to the relatively large range in the timing of filling in the questionnaire, the information on the stage of smoking, quitting and duration of smoking during pregnancy is not suitable for statistical analysis. Since main analysis is based on smoking in ‘at least the first 4 weeks of pregnancy’, infants of quitters during pregnancy were also included in the analysis, which is more likely to lead to underestimating than overestimating the effect of smoking. Sensitivity analyses on the effect of smoking intensity showed that mothers with pre-pregnancy overweight tended to have a higher smoking intensity than non-overweight mothers, but that the effect of smoking intensity on a lower birth weight in their infants was smaller. Our observation that the effects of maternal smoking and overweight on birth weight cancel each other out, may partly be due to this counterbalancing effect of smoking intensity. Thus, the net combined effect of smoking and overweight on birth weight, SGA and LGA, may be slightly different depending on smoking intensity in other study populations. Studies with larger study populations are needed to study the effect of intensity.

### Comparison with previous studies

The main effects of maternal smoking during pregnancy (158.4 g lower birth weight and an almost doubling of the risk of being born SGA compared to infants of non-smoking mothers) and maternal pre-pregnancy overweight (121.8 g higher birth weight and a 1.75 higher risk of being born LGA compared to infants of non-overweight mothers) that we observed in our study are consistent with findings reported in the literature. Lower birth weight due to maternal smoking in pregnancy may range from 150 to 300 g [2], and infants of smoking mothers have about twice the risk of being born SGA compared to those of non-smokers [16]. For maternal pre-pregnancy overweight, a recent systematic review reported that the ORs of being born LGA for infants born to overweight and obese mothers were about 1.53 (95% CI: 1.44, 1.63) and 2.08 (95% CI: 1.95, 2.23) respectively [8]. Our first sensitivity analysis shows that severity of maternal pre-pregnancy BMI did not affect birth weight in infants of mothers with both risk factors combined: these infants had similar term birth weights as infants of mothers without these risk factors. Our observation that the effects of maternal smoking during pregnancy and maternal pre-pregnancy overweight cancel each other out, is in line with the results of a large population-based study [9]. As argued above, the potential effect of smoking intensity in the association needs further study.

### Biological mechanisms and health risks

The association of lower birth weight with maternal smoking during pregnancy may be explained by many potent constituents of cigarette smoke such as carbon monoxide, polycyclic aromatic hydrocarbons and nicotine [4, 6, 17]. These toxic substances not only directly affect placental blood flow but cause the fetus to absorb these substances leading to oxygen deprivation in utero which is linked to intrauterine growth restriction, birth weight loss, and being born SGA [6, 17].

The association of higher birth weight with maternal pre-pregnancy overweight may be explained by maternal over-nutrition, although the exact mechanisms remain unclear [3, 5, 18]. Growing evidence suggests that maternal hyperglycemia is associated with metabolic and hormonal disturbances, which could result in rapid fetal growth in utero [5, 19]. For example, studies documented the link between low levels of serum adiponectin in obese mothers and increased fetal growth [20], as well as the link between impaired placental regulations caused by excess free fatty acid and fat accumulation of the fetus [21]. It is however evident that gestational weight gain independently affects infants’ birth weight and its relationship to maternal pre-pregnancy BMI is complex because not only both are closely correlated by nature (i.e. shared lifestyle factors, genetic traits) [22–24]. Since GWG is partly on the causal pathway between pre-pregnancy overweight and birth weight and since we were not able to disentangle the independent effects, we presented the main analysis without additional adjustment for GWG. Additional adjustment for GWG suggested that the effect of smoking, non-overweight women was similar. The estimates of pre-pregnancy overweight and smoking during pregnancy were somewhat stronger in the same direction as the effect estimates in the main analysis; the combined effect on birth weight was slightly more attenuated towards no effect than in the main analysis.

### Study implication

The biological pathways of maternal smoking and maternal overweight on the fetus differ and our study results do not suggest that these biological pathways interfere when both risk factors are present in pregnant women. This indicates that the fetus may be at risk of different adverse outcomes depending on whether the exposure consists of maternal smoking or maternal pre-pregnancy overweight because separate pathways are involved in the exposure-outcome relationship. For example, sudden infant death syndrome and stillbirth are attributed to maternal smoking [1, 2, 4] and congenital anomalies attributed to pre-pregnancy overweight [3, 5]. Furthermore, both maternal risk factors put the fetus at higher risk of developing diseases later in life such as cardio-metabolic diseases (i.e. overweight/obesity) in childhood as reported by earlier studies [8, 19, 25–28]. Thus, further investigation into the long term effects on child health due to the combination of both maternal risk factors is needed..

Our findings may have important implications for perinatal practice. We observed that the effects of maternal smoking during pregnancy and maternal pre-pregnancy overweight on the infant’s term birth weight cancel each other out, but the potential adverse health effects during childhood are different for each maternal risk factor and these may add up. Since the infant’s birth weight is often used as a health indicator in perinatal practice, this may mask potential health risks over the life course.

## Conclusion

Infants from mothers who smoked during pregnancy and who also had pre-pregnancy overweight had a similar term birth weight and similar risk of being SGA and LGA as infants from mothers without these risk factors. This indicates that the lower birth weight associated with maternal smoking and the higher birth weight associated with maternal pre-pregnancy overweight cancel each other out.

## Supplementary information

**Additional file 1: Table S1.** Associations of maternal smoking during pregnancy with term birth weight and the risk of being SGA; overall and in strata of pre-pregnancy overweight. **Table S2.** Associations of maternal pre-pregnancy overweight, obesity with term birth weight and the risk of being LGA; overall and in strata of maternal smoking during pregnancy. **Table S3.** The effects of the combinations of maternal risk factors on term birth weight and the risk of being SGA and LGA. **Table S4.** Smoking intensity in mothers who smoked during pregnancy by pre-pregnancy overweight status and birth weight of their infants. **Table S5a.** Association of smoking intensity and birth weight: overall and in strata of maternal pre-pregnancy overweight. **Table S5b.** Associations of maternal smoking during pregnancy with term birth weight and the risk of being SGA: overall and in strata of maternal pre-pregnancy overweight, similar to Table 2 in main paper, but excluding infants of smoking mothers with missing data on smoking intensity (121 missing). **Table S6.** The effects of the combinations of exposure to maternal smoking during pregnancy and pre-pregnancy overweight on term birth weight and the risk of being SGA and LGA.

## Data Availability

The data underlying the findings presented in this paper are available on request. Requests can be submitted to the PIAMA Principal Investigators. Their names and e-mail addresses are listed on the PIAMA website (http://piama.iras.uu.nl/index-en.php#collaboration). The PIAMA data are not freely accessible in the public domain, because this would be in conflict with the agreement between the PIAMA study team and the PIAMA participants. The information participants received at the start of the study (in 1996 ± 1997) included the statement ‘the information that we receive from you will only be used for the PIAMA project’ and participants gave written informed consent based on this information.

## Abbreviations

ANOVA: Analysis of variance
BMI: Body mass index
CI: Confidence interval
GWG: Gestational weight gain
IOM: Institution of Medicine
LGA: Large-for-gestational age
OR: Odds ratio
PIAMA: The Prevention and Incidence of Asthma and Mite Allergy Study
SGA: Small-for-gestational age
