# Supplementary material for: The joint effect of maternal smoking during pregnancy and maternal pre-pregnancy overweight on infants’ term birth weight
Source: BMC Pregnancy Childbirth. 2020 Feb 27;20:132. doi: 10.1186/s12884-020-2816-3 (PMC7047372; doi:10.1186/s12884-020-2816-3)
Supplement: Supplementary file 1 — Additional file 1: Table S1. Associations of maternal smoking during pregnancy with term birth weight and the risk of being SGA; overall and in strata of pre-pregnancy overweight. Table S2. Associations of maternal pre-pregnancy overweight, obesity with term birth weight and the risk of being LGA; overall and in strata of maternal smoking during pregnancy. Table S3. The effects of the combinations of maternal risk factors on term birth weight and the risk of being SGA and LGA. Table S4. Smoking intensity in mothers who smoked during pregnancy by pre-pregnancy overweight status and birth weight of their infants. Table S5a. Association of smoking intensity and birth weight: overall and in strata of maternal pre-pregnancy overweight. Table S5b. Associations of maternal smoking during pregnancy with term birth weight and the risk of being SGA: overall and in strata of maternal pre-pregnancy overweight, similar to Table 2 in main paper, but excluding infants of smoking mothers with missing data on smoking intensity (121 missing). Table S6. The effects of the combinations of exposure to maternal smoking during pregnancy and pre-pregnancy overweight on term birth weight and the risk of being SGA and LGA. [file 12884_2020_2816_MOESM1_ESM.docx]

**Supplementary Tables**

Table S1 Associations of maternal smoking during pregnancy with term birth weight and the risk of being SGA; overall and in strata of pre-pregnancy overweight

|  | **Birth weight (g)** | | **SGA** | | |
| --- | --- | --- | --- | --- | --- |
|  | **Crude Diff (g)**  **(95% CI)** | **Adjusted Diff ^a^ (g)**  **(95% CI)** | **SGA/Total**  **(%)** | **Crude OR**  **(95% CI)** | **Adjusted OR ^b^**  **(95% CI)** |
| **Main effect:** |  |  |  |  |  |
| Non-smoking | 0 (Ref) | 0 (Ref) | 179/2,681  (6.7%) | 1.0 (Ref) | 1.0 (Ref) |
| Smoking | -167.6  (-211.5, -123.7) | -158.4  (-203.4, -113.4) | 69/545  (12.7%) | 2.02  (1.51, 2.72) | 1.92  (1.42, 2.60) |
| **Strata of maternal pre-pregnancy overweight:** | | | | | |
| ***Non-overweight*** | |  |  |  |  |
| Non-smoking | 0 (Ref) | 0 (Ref) | 157/2,168  (7.2%) | 1.0 (Ref) | 1.0 (Ref) |
| Smoking | -177.1  (-226.6, -127.6) | -164.5  (-215.2, -113.8) | 58/418  (13.9%) | 2.06  (1.49, 2.85) | 1.96  (1.41, 2.72) |
| ***Overweight*** | |  |  |  |  |
| Non-smoking | 0 (Ref) | 0 (Ref) | 17/412  (4.1%) | 1.0 (Ref) | 1.0 (Ref) |
| Smoking | -157.8  (-260.6, -55.1) | -142.1  (-246.9, -37.3) | 8/96  (8.3%) | 2.11  (0.88, 5.05) | 2.10  (0.86, 5.12) |
| **Obesity** |  |  |  |  |  |
| Non-smoking | 0 (Ref) | 0 (Ref) | 5/101  (4.9%) | 1.0 (Ref) | 1.0 (Ref) |
| Smoking | -168.0  (-389.9, 53.9) | -163.2  (-394.1, 67.7) | 3/31  (9.7%) | 2.06  (0.47, 9.15) | 1.24  (0.26, 5.99) |

**Abbreviations:** Diff =mean difference; SGA = Small for gestational age; OR = Odds ratio; CI = Confidence interval

^a^ Multivariable linear regression model adjusted for maternal age, and education. The main effect of maternal smoking on birth weight was obtained from the model: *term birth weight = α + β(smoking) + covariates*. This model was used in stratified analyses where the adjusted coefficients represent the difference in term birth weight for infants of smoking vs non-smoking mothers within strata of maternal pre-pregnancy overweight (BMI 25-29 kg/m^2^), and maternal pre-pregnancy obesity (BMI ≥ 30 kg/m^2^).

^b^ Multivariable logistic regression model adjusted for maternal age, and education. The main effect of smoking on the risk of being SGA was obtained from the model: *logit[P(SGA)] = α + β(smoking) + covariates*. This model was used in stratified analyses where the odds ratios represent the risk of being SGA for infants of smoking vs non-smoking mothers within strata of maternal pre-pregnancy overweight (BMI 25-29 kg/m^2^), and maternal pre-pregnancy obesity (BMI ≥ 30 kg/m^2^).

**Table S2** Associations of maternal pre-pregnancy overweight, obesity with term birth weight and the risk of being LGA; overall and in strata of maternal smoking during pregnancy

|  | **Birth weight (g)** | | **LGA** | | |
| --- | --- | --- | --- | --- | --- |
|  | **Crude Diff**  **(95% CI)** | **Adjusted Diff ^a^**  **(95% CI)** | **LGA/Total**  **(%)** | **Crude OR**  **(95% CI)** | **Adjusted OR ^b^**  **(95% CI)** |
| **Main effect:** |  |  |  |  |  |
| Non-overweight | 0 (Ref) | 0 (Ref) | 261/2,586  (10.1%) | 1.0 (Ref) | 1.0 (Ref) |
| Overweight | 94.0  (48.4, 139.6) | 105.4  (59.4, 151.5) | 76/508  (14.9%) | 1.57  (1.19, 2.06) | 1.60  (1.21, 2.12) |
| Obesity | 160.4  (76.3, 244.4) | 186.6  (101.7, 271.5) | 27/132  (20.5%) | 2.29  (1.47, 3.56) | 2.41  (1.54, 3.78) |
| **Strata maternal smoking during pregnancy:** | |  |  |  |  |
| ***Non-smoking*** | |  |  |  |  |
| Non-overweight | 0 (Ref) | 0 (Ref) | 228/2,168  (10.5%) | 1.0 (Ref) | 1.0 (Ref) |
| Overweight | 95.6  (46.1, 145.0) | 102.5  (52.5, 152.5) | 68/412  (16.5%) | 1.68  (1.25, 2.26) | 1.69  (1.26, 2.28) |
| Obesity | 171.1  (77.4, 264.9) | 183.7  (89.0, 278.4) | 21/101  (20.8%) | 2.23  (1.35, 3.68) | 2.25  (1.35, 3.73) |
| ***Smoking*** | |  |  |  |  |
| Non-overweight | 0 (Ref) | 0 (Ref) | 33/418  (7.9%) | 1.0 (Ref) | 1.0 (Ref) |
| Overweight | 114.8  (3.3, 226.4) | 125.7  (12.8, 238.6) | 8/96  (8.3%) | 1.06  (0.47, 2.38) | 1.16  (0.51, 2.62) |
| Obesity | 180.2  (-4.7, 365.3) | 220.1  (31.7, 408.5) | 6/31  (19.4%) | 2.80  (1.07, 7.31) | 3.60  (1.31, 9.92) |

**Abbreviations:** Diff =mean difference; LGA = Large for gestational age; OR = Odds ratio; CI = Confidence interval

^a^ Multivariable linear regression model adjusted for maternal age, and education. The main effect of maternal pre-pregnancy overweight on birth weight was obtained from the model: *term birth weight = α + β1(overweight) + β2(obesity) + covariates*. This model was used in stratified analyses where the adjusted coefficients represent the difference in birth weight for infants of pre-pregnancy overweight, and obese vs non-overweight mothers within strata of maternal smoking.

^b^ Multivariable logistic regression model adjusted for maternal age, and education. The main effect of maternal pre-pregnancy overweight on the risk of being LGA was obtained from the model: *logit[P(LGA)] = α + β1(overweight) + β2(obesity) + covariates*. This model was used in stratified analyses where the odds ratios represent the risk of being LGA for infants of pre-pregnancy overweight, and obese vs non-overweight mothers within strata of maternal smoking.

**Table S3**The effects of the combinations of maternal risk factors on term birth weight and the risk of being SGA and LGA

| **Categories** | | **Birth weight (g)** | | **SGA** | | **LGA** | |
| --- | --- | --- | --- | --- | --- | --- | --- |
|  |  | **Crude Diff (g)**  **(95% CI)** | **Adjusted Diff ^a^ (g)**  **(95% CI)** | **Crude OR**  **(95% CI)** | **Adjusted OR ^b^**  **(95% CI)** | **Crude OR**  **(95% CI)** | **Adjusted OR ^c^**  **(95% CI)** |
| **SM -** | **NW** | 0 (Ref) | 0 (Ref) | 1.0 (Ref) | 1.0 (Ref) | 1.0 (Ref) | 1.0 (Ref) |
| **SM -** | **OV** | 95.6  (45.5, 145.7) | 104.3  (53.7, 154.9) | 0.55  (0.33, 0.92) | 0.53  (0.32, 0.89) | 1.68  (1.25, 2.26) | 1.70  (1.27, 2.29) |
| **SM -** | **OB** | 171.1  (76.1, 266.2) | 187.9  (92.2, 283.7) | 0.67  (0.27, 1.66) | 0.60  (0.24, 1.52) | 2.23  (1.36, 3.68) | 2.30  (1.38, 3.81) |
| **SM +** | **NW** | -177.1  (-226.8, -127.4) | -163.2  (-213.8, -112.6) | 2.06  (1.49, 2.84) | 1.94  (1.39, 2.69) | 0.73  (0.49, 1.07) | 0.75  (0.51, 1.10) |
| **SM +** | **OV** | -62.3  (-158.7, 34.1) | -44.8  (-142.7, 53.0) | 1.16  (0.55, 2.44) | 1.04  (0.49, 2.21) | 0.77  (0.37, 1.61) | 0.81  (0.38, 1.70) |
| **SM +** | **OB** | 3.1  (-165.7, 172.0) | 34.7  (-135.9, 205.3) | 1.37  (0.41, 4.56) | 1.13  (0.33, 3.79) | 2.04  (0.83, 5.03) | 2.17  (0.87, 5.44) |

**Abbreviations**: Diff = mean Difference; SGA = Small for gestational age; LGA = Large for gestational age; OR = Odds ratio; CI = Confidence interval; SM = Smoking; OV = Overweight; OB = Obesity; NW = non-overweight, non-obesity

^a^ Multivariable linear regression model adjusted for maternal age, and education. The main effect of each category was obtained from the model below. The adjusted coefficient represents the difference in birth weight of infants of each category of interest compared to infants of non-smoking, non-overweight *and* non-obese mothers.

*term birth weight = α + β1(non-smoking, overweight) +β2(non-smoking, obesity) + β3(smoking, normal weight) + β4(smoking, overweight) + β5(smoking, obesity) + covariates.*

^b^ Multivariable logistic regression model adjusted for maternal age, and education. The main effect of each category was obtained from the model below. The odds ratio represents the risk of being SGA for infants of each category of interest compared to infants of non-smoking, non-overweight and non-obese mothers.

*logit[P(SGA)] = α + β1(non-smoking, overweight) +β2(non-smoking, obesity) + β3(smoking, normal weight) + β4(smoking, overweight) + β5(smoking, obesity) + covariates.*

^c^ Multivariable logistic regression model adjusted for maternal age, and education. The main effect of each category was obtained from the model below. The odds ratio represents the risk of being LGA for infants of each category of interest compared to infants of non-smoking, non-overweight and non-obese mothers.

*logit[P(LGA)] = α + β1(non-smoking, overweight) +β2(non-smoking, obesity) + β3(smoking, normal weight) + β4(smoking, overweight) + β5(smoking, obesity) + covariates.*

**Table S4** Smoking intensity in mothers who smoked during pregnancy by pre-pregnancy overweight status and birth weight of their infants

|  |  | **Intensity of smoking (cigarettes/day) in mothers who smoked during pregnancy**  **(n=429) *** | | | |
| --- | --- | --- | --- | --- | --- |
|  |  | **> 0- 2**  **(n=74)** | **3-5**  **(n=141)** | **6-10**  **(n=137)** | **>10**  **(n=77)** |
| **Pre-pregnancy status** |  |  |  |  |  |
| Non-overweight  N=324 (row % ) |  | 18.8 (61) | 32.7 (106) | 31.5 (102) | 17.0 (55) |
| Overweight  N=105 (row %) |  | 12.3 (13) | 33.3 (35) | 33.3 (35) | 21.0 (22) |
| **Birth weight (gram)** | | | | | |
| Non-overweight  Mean (SD) |  | 3586 (465) | 3328 (527) | 3247 (508) | 3244 (342) |
| Overweight  Mean (SD) |  | 3548 (258) | 3562 (457) | 3306 (531) | 3370 (462) |

* missing information on number of cigarettes/day among smokers (n=121)

**Table S5a** Association of smoking intensity and birth weight*: overall and in strata of maternal pre-pregnancy overweight.

|  | **Birth weight (g)** | |
| --- | --- | --- |
|  | **Crude Diff (g)**  **(95% CI)** | **Adjusted Diff*(g)**  **(95% CI)** |
| **Main effect:** |  |  |
| 0 (non-smoking)  (n=2691) | 0 (Ref) | 0 (Ref) |
| >0-2  (n=74) | -8.6 (-117.9, 100.7) | -7.1 (-116.8, 102.5) |
| 3-5  (n=141) | -201.5 (-281.7, -121.4) | -197.6 (-278.1, -117.0) |
| 6-10  (n=137) | -325.4 (-406.7, -244.1) | -317.7 (-401.2, -234.1) |
| >10  (n=77) | -307.7 (-414.9, -200.5) | -297.5 (-407.4, -187.6) |
| **Strata maternal pre-pregnancy overweight:** | | |
| ***Non-overweight*** | |  |
| 0 (non-smoking) | 0 (Ref) | 0 (Ref) |
| >0-2 | 19.1 (-100.7, 139.1) | 21.3 (-98.9, 141.5) |
| 3-5 | -238.4 (-330.3, -146.5) | -234.6 (-326.9, -142.4) |
| 6-10 | -319.4 (-413.0, -225.8) | -304.8 (-400.9, -208.7) |
| >10 | -322.5 (-448.6, -196.3) | -306.3 (-436.3, -176.2) |
| ***Overweight*** | |  |
| 0 (non-smoking) | 0 (Ref) | 0 (Ref) |
| >0-2 | -129.2 (-389.3, 130.7) | -128.0 (-389.5, 133.4) |
| 3-5 | -115.2 (-277.0, 46.4) | -100.0 (-262.5, 62.3) |
| 6-10 | -370.7 (-532.5, -209.0) | -358.2 (-524.5, -191.9) |
| >10 | -307.5 (-509.1, -105.9) | -305.3 (-508.6, -102.1) |

**Abbreviations:** Diff =mean difference; CI = Confidence interval

* the analysis based on 3,120 infants; 2,691 infants of non-smoking mothers and 429 infants of smoking mothers who had complete information on smoking intensity. 121 infants had missing data on smoking intensity

* adjusted for maternal age and maternal education.

The effect of smoking on lower birth weight was larger in the population with data on smoking intensity (429 infants of smoking mothers and 2691 infants of non-smoking mothers) than in the population with complete data on the dichotomous smoking variable in table 2 of the main text (550 infants of smoking mothers and 2691 infants of non-smoking mothers). Therefore the main analysis with smoking as a dichotomous variable was repeated in the population with complete data on smoking intensity (Table S5b). This indicates that the effect on lower birth weight was smaller in infants who had missing data on smoking intensity. However the tendency of the association between smoking intensity and lower birth weight is demonstrated in table S5a.

**Table S5b** Associations of maternal smoking during pregnancy with term birth weight and the risk of being SGA*: overall and in strata of maternal pre-pregnancy overweight, similar to table 2 in main paper, but excluding infants of smoking mothers with missing data on smoking intensity (121 missing**).**

|  | **Birth weight (g)** | | **SGA** | | |
| --- | --- | --- | --- | --- | --- |
|  | **Crude Diff (g)**  **(95% CI)** | **Adjusted Diff ^a^(g)**  **(95% CI)** | **SGA/Total**  **(%)** | **Crude OR**  **(95% CI)** | **Adjusted OR^b^**  **(95% CI)** |
| **Main effect:** |  |  |  |  |  |
| Non-smoking  (n=2,691) | 0 (Ref) | 0 (Ref) | 179/2681 (6.7%) | 1.0 (Ref) | 1.0 (Ref) |
| Smoking  (n=429) | -226.9  (-275.3, -178.5) | -217.9  (-267.6, -168.1) | 64/425  (15.1%) | 2.48  (1.82, 3.36) | 2.34  (1.71, 3.21) |
| **Strata of maternal pre-pregnancy overweight:** | | | | | |
| ***Non-overweight*** | |  |  |  |  |
| Non-smoking | 0 (Ref) | 0 (Ref) | 157/2168 (7.2%) | 1.0 (Ref) | 1.0 (Ref) |
| Smoking | -229.7  (-284.9, -174.5) | -217.4  (-274.1, -160.7) | 53/322  (16.5%) | 2.52  (1.80, 3.53) | 2.39  (1.69, 3.38) |
| ***Overweight*** | |  |  |  |  |
| Non-smoking | 0 (Ref) | 0 (Ref) | 22/513  (4.3%) | 1.0 (Ref) | 1.0 (Ref) |
| Smoking | -242.4  (-341.9, -143.0) | -230.3  (-332.3, -128.4) | 11/103  (10.7%) | 2.67  (1.25, 5.69) | 2.39  (1.09, 5.23) |

**Abbreviations:** Diff =mean difference; SGA = Small for gestational age; OR = Odds ratio; CI = Confidence interval

* the analysis based on 3,120 infants; 2,691 infants of non-smoking mothers and 429 infants of smoking mothers who had complete information on smoking intensity.

^a^ Multivariable linear regression adjusted for maternal age, and maternal education.

^b^ Multivariable logistic regression adjusted for maternal age, and maternal education

**Table S6** The effects of the combinations of exposure to maternal smoking during pregnancy and pre-pregnancy overweight on term birth weight and the risk of being SGA and LGA.

| **Categories** | | **Birth weight (g)** | | **SGA** | | **LGA** | |
| --- | --- | --- | --- | --- | --- | --- | --- |
|  |  | **Crude Diff (g)**  **(95% CI)** | **Adjusted Diff ^a^ (g)**  **(95% CI)** | **Crude OR**  **(95% CI)** | **Adjusted**  **OR ^b^**  **(95% CI)** | **Crude OR**  **(95% CI)** | **Adjusted**  **OR ^c^**  **(95% CI)** |
| **SM -** | **OV-** | 0 (Ref) | 0 (Ref) | 1.0 (Ref) | 1.0 (Ref) | 1.0 (Ref) | 1.0 (Ref) |
| **SM+** | **OV-** | -177.1  (-226.8, -127.4) | -206.3  (-256.8, - 155.9) | 2.06  (1.49, 2.84) | 2.26  (1.62, 3.17) | 0.73  (0.49, 1.07) | 0.55  (0.36, 0.83) |
| **SM -** | **OV+** | 110.4  (64.6, 156.2) | 131.3  (85.4, 177.1) | 0.57  (0.36, 0.90) | 0.49  (0.30, 0.78) | 1.79  (1.37, 2.33) | 1.93  (1.46, 2.56) |
| **SM+** | **OV+** | -46.6  (-131.2, 38.1) | -9.90  (-95.7, 75.9) | 1.21  (0.64, 2.30) | 0.89  (0.45, 1.78) | 1.05  (0.59, 1.87) | 0.94  (0.50, 1.77) |

**Abbreviations**: Diff = mean Difference; SGA = Small for gestational age; LGA = Large for gestational age; OR = Odds ratio; CI = Confidence interval; SM=maternal smoking during pregnancy; OV = maternal pre-pregnancy overweight

^a^ Multivariable linear regression adjusted for maternal age, education and absolute gestational weight gain.

^b^ Multivariable logistic regression adjusted for maternal age, education and gestational weight gain.

^c^ Multivariable logistic regression adjusted for maternal age, education and gestational weight gain.
